# The Molecular Basis of Tolerance

**Published:** 2008

**Authors:** Andrzej Z. Pietrzykowski, Steven N. Treistman

**Keywords:** Alcohol exposure, alcohol and other drug (AOD) tolerance, biological AOD tolerance, behavioral AOD tolerance, molecular AOD tolerance, acute AOD tolerance, rapid AOD tolerance, chronic AOD tolerance, membrane channel, BK channel, epigenetics

## Abstract

Tolerance is defined as the diminished response to alcohol or other drugs over the course of repeated or prolonged exposure. This mechanism allows physiological processes to achieve stability in a constantly changing environment. The onset of tolerance may occur within minutes, during a single exposure to alcohol (i.e., acute tolerance), or over longer timeframes and with prolonged exposure to alcohol (i.e., rapid or chronic tolerance). Changes in tolerance induced by alcohol may affect several processes at the molecular, cellular, or behavioral level. These effects often are interrelated and may be difficult to separate. This article describes changes at the molecular level that are related to the onset of acute, rapid, or chronic tolerance. It focuses on neuronal membrane–bound channels and the factors that affect their function and production, such as modification of protein synthesis and activity, interaction with the membrane lipid microenvironment, epigenetic effects on cytoplasmic regulation, and gene transcription. Also considered is the genetics of tolerance.

Tolerance to a drug, including alcohol, was first described as a form of behavioral plasticity and was defined as a decreased response to repeated drug exposures (Kalant 1998). Tolerance can be described at different levels of biological complexity—molecular, cellular, and behavioral—or by its temporal characteristics of how rapidly the alcohol affects the organism.

Behavioral tolerance is measured at the level of the activity of an entire animal, resulting from mutual interactions in several brain structures and with other systems. For example, a simple walk on a straight wooden beam is a good measure of a mouse’s coordination and sense of balance. Several brain regions (e.g., visual cortex, occipital cortex, cerebellum) “talk” to each other, as well as with other systems (e.g., muscular–skeletal) to enable the mouse to successfully walk from one end of the beam to the other. Alcohol intoxication impairs this complex coordination and a “drunken” mouse falls off the beam. An alcohol-tolerant mouse can, at least partially, retain its coordination and walk on the beam almost as well as an alcohol-abstinent mouse, in spite of the ingestion of alcohol (Grieve and Littleton 1979). Previous studies by Hoffman and Tabakoff (1989) have stressed the difference between “intrinsic” tolerance, which results from changes within the neurons controlling a behavior, and “extrinsic” tolerance, which results in behavioral adaptation via alterations in compensatory neural circuits (i.e., not via molecular tolerance within the primary neural circuit).

Currently, behavioral tolerance is divided into three categories: acute, rapid, and chronic (Crabbe et al. 1979; Kalant 1998; LeBlanc et al. 1975) (figure 1). Acute tolerance develops within a single drinking session, typically within minutes, whereas chronic tolerance occurs after a longer time, usually following days of continuous or intermittent alcohol exposure. Rapid tolerance shares many similarities with chronic tolerance but develops faster, typically within 8 to 24 hours.

Cellular tolerance typically is assessed at the level of a neuronal tissue consisting of a network of many neuronal and supportive cells, or even a single neuron.

Knowledge of molecular tolerance comes from dissecting adaptational processes developed by individual molecules (e.g., ion channels) during exposure to a drug. The current notion is that even complex behavioral traits can be traced to individual molecules. All of alcohol’s complex effects on an organism start at the molecular level, during interaction of an alcohol molecule with its molecular target(s).

Although these interactions at the molecular level are complex, the level of complexity increases at the cellular and behavioral level because of multiple, complex, intertwined pathways that contribute to the development of tolerance. It still is unclear how interactions with molecules in the brain eventually can lead to the altered behavior defined as alcohol dependence. However, there is a great deal of information on the mechanism of tolerance at the molecular level; thus, this review will focus primarily on that level and discuss potential relationships with other levels, such as acute, rapid, and chronic tolerance at the behavioral level, when possible.

At the molecular level, our discussion will first address mechanisms involving modification of a mature, functional ion channel present in the neuronal plasma membrane and then move progressively upstream to the nucleus, site of the “birth” of the channel protein (see sidebar). The approach in this article will be to focus on a single channel type, the BK channel—a potassium ion channel of high conductance regulated by both voltage and calcium—that has been implicated in the onset of tolerance. This review focuses on the BK channel for several reasons: (1) This channel is abundantly expressed in many brain regions (MacDonald et al. 2006; Sausbier et al. 2006; Wanner et al. 1999); (2) it plays an essential role in many aspects of neuronal physiology, including neurotransmitter release, shaping of action potentials, and dendritic integration (Greffrath et al. 1998; Higgins et al. 2008; Knott et al. 2002; Martin et al. 2004; Meredith et al. 2006; Miranda et al. 2003); (3) its activity is regulated by alcohol (Brodie et al. 2007; Dopico et al. 1998, 1999; Harris et al. 2008; Liu et al. 2004; Pietrzykowski et al. 2004); (4) it is one of the key elements of behavioral tolerance to alcohol (at least in invertebrates) (Cowmeadow et al. 2005, 2006; Davies et al. 2003; Ghezzi et al. 2004); and (5) it is the model that the authors are most familiar with. However, whereas the focus on a single channel protein is useful, it means that significant work by many laboratories examining other alcohol targets, which might well prove equally important, is not extensively covered. When applicable, other molecules relevant to the development of alcohol tolerance are noted.

## Molecular Basis of Tolerance

Alcohol can regulate a membrane-bound ion channel in several ways: by changing the activity of proteins through posttranslational modifications, interacting with membrane lipids, interacting with auxiliary proteins, modulation of membrane protein expression (i.e., trafficking), and altering the spatial organization of membrane proteins. Alcohol’s effects on processes that regulate channel protein production, such as mRNA expression in the cytoplasm and gene expression in the nucleus, also play a role in the development of tolerance. All of these molecular processes act in concert to contribute to the development of tolerance. Nevertheless, tolerance to alcohol has been examined in a limited number of behavioral parameters (Berger et al. 2004; Davies et al. 2003; Ghezzi et al. 2004; Jorgensen et al. 1985; Scholz et al. 2000), and it is clear that the relationship between molecular mechanisms of tolerance and behavioral responses is far from understood. The following sections examine alcohol’s effects on these molecular mechanisms in greater detail.

## Effects on the Activity of Proteins

### Posttranslational Modifications

Alcohol begins to increase activity of the BK channel within seconds of exposure (Dopico et al. 1998, 1999). Current thinking is that this activation is unlikely to be mediated by second messenger^1^ systems but rather results from a direct interaction of alcohol with the channel, leading to modification of channel gating, which regulates the ratio between channel open states (during which ions flow through a channel) and closed states (during which ions cannot flow through a channel). Specifically, alcohol increases the contribution of long openings and decreases the contribution of long closures, making the BK channel more active (Dopico et al. 1996). Among the reasons for thinking that a direct interaction is likely are findings from studies in which the BK channel is isolated from its host membrane and incorporated into an artificial bilayer. When single-channel recordings then are made from this isolated channel, results indicate that alcohol is capable of modifying the open probability (an electrophysiological measurement indicating the proportion of the total recording period that a channel has been open, allowing ions to flow through it). This result indicates that alcohol acts directly on the isolated channel, increasing its potential for remaining open in a manner similar to that seen in the native membrane. However, it must be noted that an alcohol-binding site on the BK channel has not yet been identified (Harris at al. 2008). It is conceivable that proteins such as kinases and phosphatases may be carried along with the incorporated channel and that the direct action of alcohol, even in this highly reduced preparation, reflects the actions on those associated proteins. However, it also can be argued that, functionally, these enzymes are part of the “channel” complex in terms of discussing the direct effect of alcohol.

Often, this potentiation of channel opening is transient and subsides after several minutes, thus exhibiting acute tolerance (Pietrzykowski et al. 2004, 2008). This type of tolerance can be controlled by changes in the channel’s phosphorylation state (Lui et al. 2004). The phosphorylation state of any protein (including ion channels) reflects the balance between the actions of protein kinases, which transfer a phosphate group from adenosine triphosphate to the protein (i.e., phosphorylation), and protein phosphatases, which remove that group (i.e., dephosphorylation). Our recent data indicate that kinases and phosphatases are involved in alcohol potentiation of the BK channel and the development of tolerance. For example, although BK channels seem not to be constitutively phosphorylated by protein kinase A (PKA), phosphorylation by this kinase is necessary for alcohol potentiation of the BK channel. Interestingly, the additional PKA phosphorylation site present in an isoform protein of the BK channel, encoded by the alternatively spliced exon STREX, prevents potentiation by alcohol (Pietrzykowski at al. 2008). Clearly, the phosphorylation state of the BK channel is important for its sensitivity and the development of acute tolerance. Alcohol also regulates phosphorylation of other important membrane proteins, such as receptors for γ-aminobutyric acid (GABA_A_) (Harris et al. 1995; Marutha Ravindran et al. 2006) and *N*-methyl-d-aspartic acid (NMDA) (Xu and Woodward 2006). As described more extensively in recent reviews (Chandler et al. 1998; Kumar et al. 2004), alcohol-induced alterations in the phosphorylation state of these receptors contribute to the development of tolerance.

Collectively, posttranslational modifications of ion channel proteins contribute to the development of tolerance to alcohol. Ion channels, functioning in the neuronal plasma membrane, undergo phosphorylation (and dephosphorylation), which modifies their activities (see sidebar). Alcohol can affect this phosphorylation state and create a new phosphorylation set point, characteristic of alcohol-tolerant channels.

### Interaction With Membrane Lipids

In addition to alterations of the protein, such as the addition or removal of phosphate groups, more indirect effects, such as a change in the immediate lipid microenvironment, can alter alcohol pharmacology. The plasma membrane is composed of a heterogeneous population of various lipids, such as sphingomyelins, phosphatidylocholines, and cholesterol, which determine lipid bilayer thickness and rigidity. Lipid structures, which can act to segregate proteins, are microdomains called rafts (Dopico and Tigyi 2007; Morris et al. 2004). For many years, membrane lipids were thought to be the primary site of alcohol action (Chin and Goldstein 1977; Seeman 1972). More recently, however, largely because mutagenesis studies indicate that very small changes in secondary structure can lead to very large changes in alcohol sensitivity, the main focus has switched to alcohol’s direct interaction with membrane proteins.

However, emerging evidence indicates that lipids play a significant role in the response of imbedded proteins to alcohol and the development of alcohol tolerance. Altering the thickness of the lipid bilayer affects the time course of the acute response to alcohol and can completely prevent alcohol potentiation of the BK channel. BK channels embedded in a thin bilayer are strongly potentiated by alcohol, whereas channels present in a thick bilayer are inhibited (Yuan et al. 2008). Moreover, cholesterol, a main component of lipid rafts (Pike 2004), regulates BK channel sensitivity to alcohol. Increased cholesterol in the plasma membrane can diminish the alcohol potentiation of the BK channel (Crowley et al. 2003), potentially contributing to channel tolerance. Thus, the lipid microenvironment plays an essential role in the development of acute tolerance.

Both acute and chronic alcohol exposure can change lipid content—particularly that of cholesterol—in cells. Short alcohol exposure depletes cholesterol (Dolganiuc et al. 2006), presumably decreasing also the amount of plasma membrane–bound cholesterol, making membranes more fluid or disordered (Chin et al. 1978; Dolganiuc et al. 2006). In contrast, after days-long exposure to alcohol, cellular membranes become tolerant to the disordering effects of alcohol (so-called “membrane tolerance ([Chin et al. 1978; Ellingson et al. 1988]), through alterations in specific phospholipids (cardiolipin, phosphatidylinositol [Ellingson et al. 1988]) and increased cholesterol content (Chin et al. 1978; Ellingson et al. 1988).

How, exactly, membrane tolerance might contribute to behavioral tolerance is not yet clear. Nevertheless, it is possible that the altered amount of cholesterol in a microdomain surrounding the BK channel caused by either acute or chronic alcohol exposure can temper alcohol’s effect on BK channels and thus contribute to the development of acute or chronic tolerance. In summary, alcohol exposure changes the lipid composition of the plasma membrane. The new lipid environment can affect ion channel sensitivity to alcohol and contribute to either acute or chronic tolerance, or both.

**Molecular Mechanisms of Tolerance**

**Figure 1:**
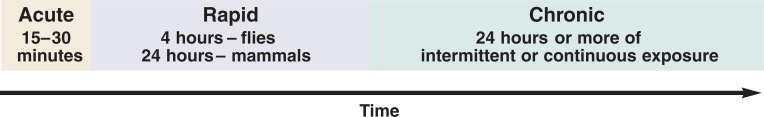
Tolerance to alcohol has been divided into three classes based on time to onset of tolerance after exposure to alcohol.

Historically, tolerance has been divided into three functional classes: acute, rapid, and chronic (see figure 1) based on how long after exposure to alcohol tolerance develops. Many molecular mechanisms underlie the development of these types of tolerance. So far, none of the known molecular mechanisms can serve as a sole marker for a particular type of tolerance. However, one can conclude that, in general, mechanisms downstream of protein synthesis contribute to acute tolerance in mammals and fruit flies (A), whereas mechanisms upstream of protein synthesis are involved in chronic tolerance in both types of organisms (C). A special case is rapid tolerance (R), which in invertebrates (fruit flies) is protein synthesis independent and in vertebrates (mammals) seems to be dependent (R*). This sidebar describes which molecular mechanism could underlie which type of tolerance, pointing to some unexplored possibilities (depicted by question marks).

**Figure:**
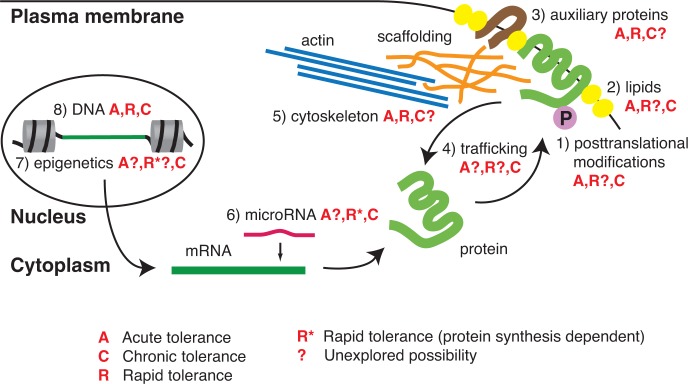

1. Posttranslational modifications of existing channels (mainly phosphorylation) have been clearly indicated to play a role in acute and chronic tolerance. However, because new channel isoforms with novel phosphorylation profiles can be created by protein synthesis–dependent mechanisms (or new kinases and/or phosphatases can be synthesized), it will be of interest to determine whether posttranscriptional mechanisms could contribute also to the development of rapid tolerance in mammals.
2. Interactions with the lipid microenvironment are important for development of acute and chronic alcohol tolerance but have not been investigated in rapid tolerance.
3. Interactions with auxiliary proteins (e.g., regulatory subunits) of channels have been shown to contribute to acute and rapid tolerance. It is highly probable that, because the development of chronic tolerance is protein synthesis dependent, changes in de novo– synthesized auxiliary proteins can add to the development of chronic tolerance.
4. Trafficking of channels to and from the cell surface has been implicated in chronic tolerance.
5. Cytoskeleton reorganization is important for the development of acute tolerance as well as rapid tolerance in fruit flies. It would be of great interest to explore whether protein synthesis–dependent types of tolerance can rely, at least partially, on cytoskeletal alteration.
6. Epigenetic cytoplasmic mechanisms (e.g., microRNA regulation) of channel message stability are important before protein synthesis from the altered transcripts has occurred, contributing to chronic or rapid tolerance in mammals.
7. Epigenetic nuclear mechanisms (e.g., chromatin remodeling) have been implicated in gene expression and subsequent de novo protein production of the fruit fly homolog of the BK channel, thus contributing to the development of chronic tolerance. However, because epigenetic changes can be, by definition, heritable, it will be of interest to determine whether both epigenetic mechanisms evoked by alcohol can contribute to any type of tolerance in offspring.
8. Genetic factors have been found to play important roles in all classes of tolerance in both invertebrate and vertebrate models.

—Andrzej Z. Pietrzykowski, M.D., Ph.D., and Steven N. Treistman, Ph.D.

### Interaction With Auxiliary Proteins

BK channels, like many other membrane proteins, are clustered with other (auxiliary) proteins (Alioua et al. 2008; Jo et al. 2005; Loane et al. 2006; Zhou et al. 2003*a*,*b*) within lipid rafts (Noble et al. 2006; Weaver et al. 2007). BK channels also can form functional dyads with voltage-dependent calcium channels (Grunnet and Kaufmann 2004; Marrion and Tavalin 1998) and bind to a multifunctional scaffolding protein known as RACK1 (Isacson et al. 2007), or β2 adrenergic receptors (Liu et al. 2004). Auxiliary β-subunits are tightly bound proteins that affect BK channel characteristics such as its sensitivity to calcium, an important molecule in cell-to-cell communication (see sidebar). Recently, it was discovered that β-subunits of the BK channel also can control the alcohol response of these channels and influence the development of tolerance. For example, upon cotransfection of HEK293 cells with both the BK channel main α-subunit and an auxiliary β1-subunit, the resulting BK channel is composed of α-β1 complexes. The association of the β1-subunit with the α-subunit prevents the potentiation of the channel by alcohol (Martin et al. 2004). Interestingly, the β4-subunit also reduces this potentiation (Feinberg-Zadek and Treistman 2007; Martin et al. 2004). The molecular mechanism of β-subunit regulation of BK channel alcohol sensitivity remains unclear. Expression of BK β-subunits varies among brain regions. BK β4-subunits are abundant throughout the brain, whereas expression of BK β1-subunits is, in general, very low (Martin et al. 2004).

Recent studies indicate that the BK β4-subunit exerts a profound influence on the development of acute tolerance. BK channels from wild-type mice show little alcohol tolerance compared with channels from BK β4-knockout mice, which exhibit acute tolerance. Moreover, BK β4’s role in the development of tolerance also can be observed on a level of neuronal activity. BK channels contribute to the frequency of electrical signals (i.e., action potentials, also known as spikes) generated by neurons. Alcohol persistently decreases the number of action potentials in striatal slices harvested from wild-type mice. However, the change in action potential frequency lasts only a few minutes in the same preparation harvested from BK β4-knockout mice. This transient change in spike patterning is consistent with the development of tolerance. β4-knockout mice also develop behavioral rapid tolerance to the locomotor effects of alcohol, in contrast to the wild-type mice. Most striking, the β4-knockout mice consume significantly greater amounts of alcohol, consistent with human studies, which indicates a correlation between the degree of acute behavioral tolerance and the likelihood of future alcohol abuse (Martin et al. 2008).

Changes in subunit composition as a potential mechanism in the development of tolerance is not limited to the BK channel. It is well established that alcohol selectively alters mRNA and protein expression of selected subunits of membrane proteins such as NMDA and GABA receptors. For example, chronic alcohol exposure selectively increases expression of the NMDA receptor R2B subunit protein and subsequently increases its surface expression and targeting to synapses (Follesa and Ticku 1995; Hu and Ticku 1995; Qiang et al. 2007; Senatorov et al. 1995). This mechanism contributes to the alcohol-triggered upregulation of NMDA receptor function (Follesa and Ticku 1996) and the development of alcohol tolerance (Faingold et al. 1998). Similarly, chronic alcohol exposure results in the reorganization of GABA_A_ receptor subunits, such as the downregulation of GABA_A_ receptor δ(Mehta et al. 2007) and α1-subunits (Cagetti et al. 2003) or the down- or upregulation of the α4-subunit (depending on its neuronal location [Cagetti et al. 2003]). These changes, in turn, contribute to GABA_A_ receptor–related alcohol tolerance (Cagetti et al. 2003).

From the allostatic point of view (see discussion of the concept of allostasis in the textbox), modulation of specific subunits of BK, NMDA, and GABA_A_ receptors (and probably other channels and receptors as well) establishes new expression levels of these subunit proteins. To maintain channel function, new “equilibria” of subunits within a channel are formed, allowing “stability through change.” Such modified channel subunit composition contributes to the development of tolerance.

Can it be stated that allostatic changes in subunit composition of ion channels contribute selectively to acute, rapid, or chronic types of tolerance? Most experiments to date have been performed using alcohol exposures lasting several days, thus relating change in subunit composition to chronic tolerance. However, there is some evidence indicating that subunit reorganization can be rapid. Recent data by Liang and colleagues (2007) demonstrate that as early as 1 hour after alcohol exposure, cell surface expression of GABA_A_ receptor auxiliary subunits α4 and δ(but not others) is significantly decreased. These results suggest that alcohol-triggered reorganization of channel subunits also could contribute to acute tolerance. Further studies determining the role of auxiliary proteins in other types of tolerance are warranted.

### Modulation of Membrane Protein Expression

Alcohol’s modulation of channel cell surface expression is another molecular mechanism contributing to the development of tolerance. In neurons, the surface expression of functional membrane proteins determines neuronal properties and is highly regulated through several mechanisms. The density of channels on the neuronal surface is a precisely controlled balance among delivery of newly produced channels, recycling, internalization, and degradation.

Similar alcohol regulation of the internalization of GABA_A_ receptors in neurons has been reported. Chronic alcohol exposure causes increased internalization of GABA_A_ receptors (Kumar et al. 2003).

To fully understand the contribution of altered trafficking of channels and receptors to a particular class (e.g., acute, chronic) of tolerance, it will be essential to determine whether these alterations are protein synthesis dependent. Some hints are coming from a recent BK channel gene regulation study (Pietrzykowski at al. 2008) indicating that alcohol invokes changes in BK mRNA level, which precede altered trafficking of the channel (Pietrzykowski at al. 2004).

### Spatial Organization of Membrane Proteins

Some channel types (such as BK) are not distributed homogeneously on the neuronal surface but occur in smaller clusters of BK channels, or microdomains. It has been observed that alcohol alters the cell surface expression of BK channels in neurons, similar to a mechanism of tolerance reported for other drugs (Pietrzykowski et al. 2004). In presynaptic terminals, where BK channels are expressed in clusters, alcohol causes declustering of BK channels in the plasma membrane and redistribution of channels between the membrane and cytoplasm (observed as decreased channel density in the plasma membrane and increased density of channels in the terminal interior). These morphological changes are concurrent with a loss of BK alcohol sensitivity and/or tolerance (Pietrzykowski et al. 2004). Interestingly, chronic alcohol exposure also can enhance clustering of some other membrane proteins. The expression of NMDA receptor type 1 and 2B clusters is increased on the surface of cortical neurons (Qiang et al. 2007), as well as hippocampal neurons, specifically in synaptic boutons (Carpenter-Hyland et al. 2004).

Spatial organization of membrane proteins (clustering) is mediated by scaffolding proteins, which link them to specific signaling cascades and other cytoskeletal elements. Scaffolding proteins also are involved in the development of alcohol tolerance. For example, it has been shown that the scaffolding protein RACK1 organizes NMDA receptor complexes (Yaka et al. 2002) and plays an important role in the development of acute alcohol tolerance of these receptors (Yaka et al. 2003). Acute alcohol exposure inhibits NMDA receptor activity (Lovinger et al. 1989, 1990). Additionally, alcohol disrupts NMDA receptor binding to RACK1, allowing for subsequent phosphorylation of the NR2B subunit of the NMDA receptor by Fyn kinase. This phosphorylation gradually reduces alcohol- induced inhibition of the NMDA receptor, thus contributing to the development of acute tolerance (Yaka et al. 2003).

Homer, another neuronal scaffolding protein recently implicated in the development of alcohol tolerance (Urizar et al. 2007), has been shown to play a role in synapse formation, neuronal excitability (Sala et al. 2003), and addiction-related neuroplasticity (Szumlinski et al. 2008). Homer proteins form tetramers, in which four products of Homer mRNA (each called a monomer) create a single functional structure. Each Homer tetramer provides a cytoplasmic framework for receptors and channels on the plasma membrane and links them to important components inside the cell, such as intracellular messenger systems and other scaffolding proteins (Duncan et al. 2005; Sala et al. 2001). They also are part of the NMDA receptor complex (Shiraishi et al. 2003; Xiao et al. 2000), and deletion of *Homer2* decreases the amount of NMDA NR2B subunit on the cell surface (Szumlinski et al. 2005; Urizar et al. 2007).

**Allostasis**

Conceptually, any type of tolerance, as an adaptational mechanism, can be seen as the maintenance of homeostasis, initially perturbed by drug exposure. However, a simple change in homeostatic state cannot sufficiently explain a number of key elements of the role of tolerance in addiction (Koob 2003; Koob and Le Moal 2001). Thus, researchers in the addiction field have applied a different conceptual framework to the development of tolerance, called allostasis, which represents an evolution of the idea of homeostasis (Koob and Le Moal 1997).

The concept of allostasis was formulated in the late 1980s to explain basic physiological mechanisms underlying human epidemiological findings (Sterling and Eyer 1988) and then extended to include stress and anxiety (McEwen 1998; Schulkin et al. 1994). It defines physiological processes required to achieve stability in a constantly changing environment. Recently, the concept of allostasis has been used to interpret reactions to exposure to drugs of abuse (Koob and Le Moal 2001). From the homeostatic point of view, tolerance to a drug is seen as an adaptive mechanism attempting to restore normal reward function. In contrast, allostasis is the process of maintaining stability of the reward function through constant changes (“stability through change”) in the reward circuitry in the face of factors such as stress (Koob 2008; Koob and Moal 2001). A chronic deviation from homeostasis and establishment of new set points (allostatic state) is believed to play a fundamental role in the transition from normal function of the brain reward circuitry to its “dysregulation” in drug dependence and addiction (Koob and Moal 2001). Multiple, frequent drug exposures cause a progressively diminishing response to a drug (i.e., the development of tolerance). This contributes to a shift in the allostatic set point and subsequently to the development of drug dependence. Because allostatic principles may allow for a better understanding of the relationship between alcohol tolerance and dependence, it is useful to place the molecular mechanisms of tolerance within the context of allostasis.

The role of Homer in alcohol tolerance has been suggested by recent fruit fly (*D. melanogaster*) studies (Urizar et al. 2007). Mutant flies without functional Homer do not develop behavioral rapid tolerance, indicating an essential role for this protein in this particular type of tolerance. Moreover, rapid tolerance is “rescued” (meaning the ability to develop tolerance is maintained) by expression of wild-type Homer in neurons, specifically in a subset of neurons in the ellipsoid body located in the head, which are involved in higher control of locomotor activity such as walking and leg coordination (Martin et al. 1999). The role of Homer in other classes of tolerance has not yet been determined. An especially important research goal would be to determine whether similar mechanisms might be observed also in the mammalian system.

RACK1 and Homer proteins are scaffolding elements and, as such, are tightly bound to the actin cytoskeleton (Fagni at al. 2002; Shiraishi et al. 1999; Usui et al. 2003; Won et al. 2001). Actin fibers form a complex meshwork in the cell and constantly and quickly are remodeled in response to internal and external cues (Ethell and Pasquale 2005). Altered actin dynamics recently have been linked to alcohol tolerance (Offenhauser et al. 2006). In wild-type mice, acute alcohol exposure causes significant loss of filamentous actin in neurons (Offenhauser et al. 2006), presumably by targeting epidermal growth factor receptor pathway substrate (Eps8), a member of the protein family regulating actin polymerization. This alcohol-related loss of actin can be reversed quickly by washing the drug away (Offenhauser et al. 2006). In animals lacking Eps8, actin stability is enhanced, causing resistance to the destabilizing effects of alcohol. Additionally, Eps8 association with the NMDA receptor complex is thought to play a role in the development of molecular acute tolerance, as well as behavioral acute tolerance to the intoxicating effects of alcohol (Offenhauser et al. 2006). An obvious question is how these effects on actin contribute to rapid or chronic tolerance in mice.

To date, molecular mechanisms regulating protein cell surface expression seem to be involved in the development of specific classes of alcohol tolerance (i.e., rapid tolerance is linked to the Homer protein; acute tolerance to actin and RACK1). More research is needed to determine whether these molecules also contribute to other classes of tolerance and if they are molecular markers for specific classes of tolerance.

## Effects on Protein Dynamics

### Epigenetics

Epigenetics refers to heritable changes in gene expression without alterations in the DNA sequence. In addition to alcohol’s more direct effects on membrane and associated proteins, it also can affect upstream processes controlling channel production (namely, transcription and translation), including epigenetic influences on gene expression. Several molecular mechanisms of epigenetic regulation of gene expression exist. Some epigenetic mechanisms take place in the nucleus (DNA methylation, posttranscriptional modifications of histones, chromatin remodeling), whereas others occur in the cytoplasm (mRNA processing). There is growing evidence that at least some of these epigenetic mechanisms are involved in the development of tolerance, as described below.

### Epigenetic Cytoplasmic Mechanisms

Alcohol is known to modify mRNA expression of many different proteins (Hoffman and Tabakoff 2005; Kerns and Miles 2008), including the BK channel pore-forming α-subunit (Pietrzykowski et al. 2008). Typically, the α-subunit of the BK channel undergoes alternative splicing, giving rise to a number of final transcripts varying in their exonal structure (Fury et al. 2002). Researchers recently have discovered that different BK channel transcripts encode channels with different alcohol sensitivities (Kerns and Miles 2008; Pietrzykowski et al. 2008). BK channels containing an insert encoded by an alternatively spliced exon called ALCOREX are highly potentiated by alcohol, whereas others containing an insert encoded by an alternatively spliced exon called STREX are insensitive to alcohol (Pietrzykowski et al. 2008). Pietrzykowski and colleagues (2008) found that alcohol causes remodeling of the BK channel transcript landscape by regulating the stability of a subset of these transcripts, such that the relative number of transcripts encoding highly sensitive channels are decreased, whereas that of transcripts encoding less sensitive channel proteins are increased, consistent with tolerance. Differential regulation of transcript stability occurs via a microRNA-based pathway.

MicroRNAs are recently discovered, small, noncoding RNA molecules regulating mRNA and protein expression (Ambros 2001, 2004). Alcohol exposure specifically upregulates one microRNA species, miR-9, which subsequently binds to selected BK mRNA transcripts containing a miR-9–binding site in their 3 -untranslated region (3 -UTR) (Pietrzykowski et al. 2008). This binding destabilizes transcripts with miR-9 complementarity and causes their degradation. By this mechanism, the number of transcripts is reduced and their absolute and relative amounts are altered. The overall effect is a change in the contribution of each transcript product to BK channel assembly, causing an increase in the representation of alcohol-tolerant channels.

Thus, alcohol regulates gene expression using a microRNA-based mechanism. For the outcome of this regulation to be evident, new protein must be translated from modified transcripts, and delivered to its functional home. Because this process will take time, this mechanism is unlikely to contribute to acute tolerance and is more likely to play a role in rapid and chronic tolerance, which are both protein synthesis dependent in mammals. Contribution of this epigenetic mechanism to protein synthesis–independent types of tolerance, like acute or rapid tolerance in flies, is unlikely.

Alcohol regulation via a miR-9–dependent mechanism has the potential for a wide-ranging role in plasticity because many potential miR-9 targets also are regulated by alcohol (Pietrzykowski et al. 2008). The role of other small, noncoding RNAs in alcohol tolerance has not yet been addressed. However, there is growing evidence that many other microRNA species also are regulated by alcohol (Sathyan et al. 2007). Thus, further studies are warranted to determine the important role of these newly appreciated regulatory molecules in the development of alcohol tolerance.

#### Epigenetic Nuclear Mechanisms

Epigenetic nuclear mechanisms also have been implicated recently in the development of alcohol tolerance, using a fruit fly homolog of the BK channel (*Slowpoke, Slo*) as a model (Wang et al. 2007). Gene transcription is regulated by chromatin structure via posttranslational modifications (e.g., acetylation) of chromatin core proteins known as histones. Acetylation of histones weakens the association between histones and DNA, allowing binding of a transcriptosome complex to gene promoter regions, and subsequent transcription (Gregory et al. 2001).

Brief exposure to alcohol causes increased *Slo* gene expression in the central nervous system of *Drosophila* (Cowmeadow et al. 2006). Enhanced expression of the *Slo* gene is mediated by increased histone acetylation within the *Slo* promoter region. Blocking of histone deacetylation by inhibition of histone deacetylase has a similar effect, confirming the involvement of this epigenetic pathway in the regulation of *Slo* gene expression. This mechanism exposes the *Slo* promoter to the cAMP response element-binding protein (CREB). CREB binding to the *Slo* promoter region enhances *Slo* gene transcription. Thus, activation of this pathway leads to changes in *Slo* protein expression, which contributes to the development of tolerance (Wang et al. 2007). Change in *Slo* expression can be seen as an attempt to maintain “stability through change” in accord with an allostatic mode of adaptation.

### Genetics of Tolerance

Invertebrate models also are extensively used to determine links between genes and behavior, including the development of tolerance to alcohol. The *Slo* gene is one of the best examples of this link. It has been shown in both fruit fly and worm (*C. elegans*) that the behavioral response to alcohol is modified by targeting *Slo* channel function. In *Drosophila*, overexpression of *Slo* in neurons produces a phenotype mimicking alcohol tolerance. A neuron-specific loss-of-function mutation of *Slo* produces fruit flies unable to develop rapid tolerance to alcohol (Cowmeadow et al. 2005, 2006; Ghezzi et al. 2004). Similar observations have been made in worms in *Slo’s* acute response to alcohol (Davies et al. 2003). These results suggest that the establishment of a new set point for *Slo* channel expression is important in the development of tolerance to alcohol.

Fruit fly studies provide evidence for the involvement of a number of molecules and systems in the development of tolerance. Fruit flies containing a loss-of-function mutation in the gene encoding tyramine β-hydroxylase are unable to synthesize octopamine (an invertebrate analog of vertebrate’s noradrenaline) and have a diminished ability to develop rapid tolerance to alcohol (Scholz et al. 2000). Therefore, these results suggest the involvement of the noradrenergic system in the development of tolerance. Indeed, researchers have suggested a role for the adrenergic system in alcohol tolerance in mammals. In mice, the destruction of noradrenergic systems in brain by intraventricular injection of 6-hydroxydopamine prevents the development of chronic tolerance to alcohol (Ritzmann and Tabakoff 1976; Tabakoff and Ritzmann 1977; Tabakoff et al. 1977, 1986). Interestingly, mutant flies with defective biosynthesis of octopamine display reductions in rapid tolerance only, whereas chronic tolerance is unchanged (Berger et al. 2004).

A newly described gene (*Hangover*) has been found to contribute to the development of rapid tolerance to alcohol in fruit flies (Scholz et al. 2005). *Hangover* loss-of-function mutant flies show significantly reduced tolerance to alcohol compared with wild-type flies. Alcohol does not change *Hangover* expression. The exact role of the *Hangover* protein is unknown, although the predicted *Hangover* protein structure suggests its role in transcription. Thus, it is possible that alcohol, via the *Hangover* protein, can modulate transcription of other genes involved in the development of tolerance to alcohol. *Hangover* mutant flies also are defective in their response to environmental stress, supporting an important role for stress in the development of alcohol addiction and tolerance (see recent review by Koob 2008). Recent genetic studies suggest a role for *Hangover* in the development of alcoholism in humans. A single-nucleotide polymorphism in the human ortholog of *Hangover* (ZNF699) recently was correlated with alcohol dependence in Irish populations (Riley et al. 2006).

Other fruit fly screens point to additional genes and pathways that can contribute to the development of alcohol tolerance (Berger et al. 2008). Berger and colleagues (2008) screened 60 long-term memory mutants and discovered that several of these mutants display reduced rapid tolerance, others show decreased chronic tolerance, and one mutant demonstrates increased chronic tolerance.

New data also are coming from vertebrate studies providing insights into genetic influences on alcohol tolerance and also indicating that different types of tolerance can be genetically determined. Recent microarray analysis has identified at least eight candidate genes important for the development of acute tolerance to alcohol using inbred strains of mice (Hu et al. 2008). Moreover, another mouse study (Radcliffe et al. 2006) suggests that genetic factors influencing acute tolerance can play a major role in the development of rapid tolerance. Finally, successful breeding of mice displaying high and low rapid tolerance provided evidence for both a genetic contribution to rapid tolerance and for a genetic link between rapid and chronic tolerance (Rustay and Crabbe 2004). Further elucidation of these types of genetic influences underlying alcohol-relevant phenotypes may help to define the molecular elements of allostasis important in the development of different classes of tolerance.

## Summary

Work from a number of laboratories has highlighted novel molecular mechanisms underlying the development of tolerance to alcohol. An emerging model indicates that multiple, complex, and intertwined molecular pathways contribute to the development of different classes of tolerance to alcohol.

Tolerance typically is divided into three functional categories (acute, rapid, and chronic), but determining precisely which molecular underpinning underlies which class of tolerance (or if they are exclusive) can be difficult. There are multiple steps that can influence the response of surface proteins to alcohol, from gene transcription in the nucleus, to cytoplasmic regulation, to final expression in the plasma membrane. These steps are interlocked and sometimes difficult to separate and should be seen as a “continuum” rather than completely independent entities. As described here, all of these steps, including posttranslational modifications of proteins existing in the plasma membrane, their interactions with the lipid microenvironment, association with auxiliary proteins and cytoskeleton, trafficking, regulation of mRNA stability, and epigenetic and genetic mechanisms, contribute to the development of tolerance (see the sidebar for an outline of these mechanisms). Many more experiments need to be performed to determine the exact molecular mechanisms of each type of tolerance to alcohol and ultimately to define a molecular switch (or switches) allowing for transition from one type of tolerance to another. So far, one general conclusion seems to be that mechanisms which do not involve protein synthesis are related to acute tolerance in mammals and acute and rapid tolerance in fruit flies, whereas mechanisms upstream of protein synthesis are involved in rapid tolerance in mammals and in chronic tolerance in both mammals and fruit flies.

Importantly, each of these molecular mechanisms can be envisioned to contribute to a well-balanced set point according to allostatic principles. Alcohol exposure creates a new balance at each step, maintaining “stability through change.” It is likely that alcohol can trigger these mechanisms simultaneously and that the central nervous system adapts to alcohol by dynamic interaction of these mechanisms. This integrated reaction provides the molecular bases for allostatic changes associated with the development of alcohol tolerance.

The molecular underpinnings of tolerance described in this review, including membrane lipids, regulatory RNA molecules, and channel protein subunits, may provide potential therapeutic targets for alcoholism treatment. However, a rational approach to drug development will be facilitated by a clearer understanding of the relationship between molecular tolerance and the elements of addiction, such as dependency and craving, which have not been addressed in this review.

## Footnotes

1 For the definition of this and other technical terms, please see the Glossary, pp. 345–347.
